# Management and co-morbidities in children and adolescents with attention deficit hyperactivity disorder diagnosis: A clinical audit in the Eastern Cape province, South Africa

**DOI:** 10.4102/sajpsychiatry.v32i0.2585

**Published:** 2026-01-13

**Authors:** Sivuyisiwe Mpondo, Wendy Vogel, Isabel A. Michaelis

**Affiliations:** 1 Department of Paediatrics, Faculty of Medicine and Health Science, Walter Sisulu University, East London, South Africa; 2 Division of Child & Adolescent Psychiatry, University of Cape Town, Cape Town, South Africa

**Keywords:** ADHD, management, co-morbidities, low resource setting, children, adolescent

## Abstract

**Background:**

Attention deficit hyperactivity disorder (ADHD) is a common childhood disorder, with a prevalence rate of 5% – 8%. Clinical practice guidelines have been developed internationally to standardise the care and management of patients with ADHD.

**Aim:**

To assess compliance with clinical guidelines on the management of children with ADHD in two hospitals in the Eastern Cape province using the National Institute for Health and Care Excellence (NICE) guidelines as the gold standard.

**Setting:**

Paediatric departments of two hospitals in the Buffalo City Municipality, Eastern Cape province, South Africa.

**Methods:**

A clinical audit was conducted on children and adolescents diagnosed with ADHD attending neurodevelopmental clinics (NDCs). Patient folders were reviewed between June 2021 and December 2021, and data were collected using a 16-point audit tool based on the NICE guidelines for ADHD.

**Results:**

A total of 111 patient folders met the inclusion criteria. Of the 16 audit standards, 8 demonstrated over 80% compliance, while 2 achieved fair compliance (50% – 79%). Significant gaps were identified in the 6 standards with poor compliance (< 50%). Co-morbidities were highly prevalent, with 83% of patients presenting with at least one co-existing condition.

**Conclusion:**

The audit demonstrated overall good clinical compliance with the NICE guidelines for ADHD management, but it also exposed gaps in psycho-social interventions, caregiver support and the availability of structured support groups.

**Contribution:**

This study highlights service gaps in resource-limited settings and is expected to further inform government policy planning in developing auxiliary services and multidisciplinary support for children with ADHD and their families in such settings.

## Introduction

Attention deficit hyperactivity disorder (ADHD) is a common neurodevelopmental disorder of childhood, with a reported prevalence of 5% – 8%.^1,2^ It can lead to significant short- and long-term challenges, often impairing emotional well-being, academic performance and social functioning.^3,4^ Attention deficit hyperactivity disorder frequently persists into adulthood, and early diagnosis combined with evidence-based interventions can significantly improve developmental trajectories and long-term outcomes.^5,6^

The Diagnostic and Statistical Manual of Mental Disorders, Fifth Edition (DSM-5) outlines the core symptoms of ADHD as age-inappropriate patterns of inattention, hyperactivity and impulsivity that interfere with functioning.^2,7,8^ Before confirming the diagnosis, other potential causes for the presenting behaviour should be excluded.^9,10^ Diagnostic accuracy relies heavily on collateral information gathered from multiple sources, including parents, caregivers, teachers and occasionally siblings.

Numerous studies have demonstrated a strong association between ADHD and various co-morbid psychiatric and neurodevelopmental disorders.^2,11,12^ The most reported co-morbidities include autism spectrum disorder (ASD), learning disabilities, depression, anxiety disorders and externalising disorders such as oppositional defiant disorder (ODD) and conduct disorder.^11,12,13,14^ A 2019 review on children with ADHD and co-morbidities reported that intellectual disability occurred in 10% – 90%, ASD in 59% of cases, tic disorders in 55% and both depression and conduct disorder in approximately 50% of children with ADHD. In comparison, an audit conducted in Denmark in 2014 found that 52% of children with ADHD had at least one co-morbid condition, with conduct disorders being the most frequent, occurring in 16.5% of cases.^15,16^

Guidelines on the diagnosis and management of ADHD in children and young people – previously primarily from high-income countries – now include a newly published national guideline from South Africa, released in 2025.^2,17,18,19^

Research on ADHD within the African continent remains limited and under-represented in the global literature.^20,21,22^ This is particularly evident in rural areas, where access to healthcare professionals trained in the assessment and management of childhood behavioural and psychiatric disorders is severely limited.^23^ A high percentage of children in South Africa have also been found to be exposed to poverty, as well as to sexual and physical violence, increasing the likelihood of developing mental health problems.^24,25^

### Background

The audit was conducted in the neurodevelopmental clinics (NDCs) of two public hospitals in Buffalo City Municipality. Together, these hospitals serve a large catchment area with a population of approximately 2.5 million people, the majority of whom come from socio-economically disadvantaged backgrounds.

There has been a marked increase in the number of prescriptions for methylphenidate (MPH), the first-line pharmacological treatment for ADHD. Specifically, the use of the 10-mg tablet has tripled, while the use of the 20-mg capsule has doubled over an 8-year period, as reported by the pharmacy department at the urban hospital (comparing data from January 2012 to January 2020).

The NDC of the urban hospital is managed by paediatricians who have no formal training in child psychiatry. However, they regularly attend courses and conferences on the treatment and management of ADHD in children and have been running the clinics for many years. Children are referred to the NDCs from various sources, including hospitals and clinics, teachers, social workers and self-referrals from parents. Initially, junior doctors, such as medical officers and paediatricians in training, see these patients in the paediatric outpatient clinic to rule out other obvious medical conditions. Following this, a referral to the NDC for further evaluation and management is made.

A weekly script clinic is held in the urban hospital, for the renewal of MPH prescriptions, as the prescription of MPH requires a monthly signed renewal, in accordance with regulations set by the South African health authorities. Folders here were collected over a period of 6 months; thus, every child receiving medication has been captured, even if they missed one or two medication repeats. At the peri-urban Hospital, the folders were collected at the hospital’s pharmacy twice a week over the same 6-month’s period, as there is no specific script renewal clinic in that paediatric department.

### Study design and study population

A clinical audit was conducted at the two hospitals from June 2021 to December 2021. All available patient folders for children attending the NDCs were reviewed, and those meeting the inclusion criteria for ADHD were assessed for compliance with the National Institute for Health and Care Excellence (NICE) guidelines for the management of ADHD.

The assessment and management practices of clinicians caring for children diagnosed with ADHD were evaluated using an audit tick sheet. This audit instrument was adapted from a 16-item binary questionnaire originally developed by researchers from the Division of Child and Adolescent Psychiatry, University of Cape Town.^21, p. 86^ Please see Table 1 for the auditing tool used. Inclusion criteria comprised of children between 6 years of age and 17 years of age, diagnosed with ADHD, who were attending the NDC.

**TABLE 1:** Audit tool.

Clinical variables		Yes	No
**Core audit standards**			
1.	Suitably qualified ADHD diagnostician		
2.	DSM-V criteria documented		
3.	Specification of ADHD severity		
4.	Full history and physical exam before diagnosis		
5.	Assessment of possible co-morbidities		
6.	Comprehensive treatment plan (including psychological/behavioural and educational interventions)		
7.	MPH offered as appropriate first-line therapy		
8.	Referral to ADHD parent group		
9.	Written psycho-education offered to patients		
10.	Written psycho-education offered to caregivers		
**Additional audit standards**			
11.	Patient, age, gender		
12.	Point of view of child documented		
13.	Contact with teacher		
14.	Growth chart plotting and monitoring		
15.	Side effect monitoring		
16.	Treatment response monitoring on standard scales		

*Source*: Vrba K, Vogel W, de Vries PJ. Management of ADHD in children and adolescents: clinical audit in South African setting. J Child Adolesc Ment Health. 2016;28(1):1–19

ADHD, attention deficit hyperactivity disorder; DSM-V, Diagnostic and Statistical Manual of Mental Disorders, 5th Edition; MPH, methylphenidate.

### Statistical analysis

#### Data entry and analysis

Anonymised data were exported from Excel to R (Version RStudio 2022.02.0+443) for statistical analysis. Descriptive statistics were used to summarise the data. Parametric

continuous variables were reported as means with standard deviations (s.d.), while categorical variables were presented as frequencies and percentages.

#### Audit standards

The audit standards were divided into 10 core standards and 6 additional standards, in accordance with the NICE guidelines (see Table 1). For each of the 16 recommended points, adherence to the standard was recorded as ‘yes’ if the practice met the criterion, or ‘no’ if it did not. Ideally, 100% compliance with each audit criterion would be the target. However, following the approach used in the Cape Town Red Cross War Memorial Children’s Hospital audit, a ‘traffic light’ system was applied. If a criterion was met in more than 80% of cases, a green code was assigned, indicating good compliance. If the criterion was met in 50% – 79% of cases, an amber code was assigned, signifying fair compliance. Finally, if a criterion was met in less than 50% of cases, a red code was assigned, indicating poor compliance.

### Ethical considerations

Ethical approval to conduct this study was obtained from the Walter Sisulu University (No. 021/2021).

## Results

### Demographic data

A total of 125 patient folders were reviewed. Of these 125 folders, two were excluded because the patients were over the age of 17, and 12 were excluded as either duplicate folders or empty folders with no information besides the script. As folders were regularly lost, completely new folders were created, allowing patients to receive their medication on the day they arrived at the hospital.

This resulted in 111 folders being included in the final analysis. Among these, 94 folders (85%) were from the urban hospital and 17 (15%) from the periurban hospital. Most participants were male (86%), while 16 (14%) were female. The mean age was 10 years and 9 months, with a s.d. of 3 years and 2 months. The boys’ mean age was 11.0 years, while the girls’ mean age was 9 years and 3 months. In all, 74 (67%) patients were between the ages of 6 years and 11 years, and 37 (33%) were between the ages of 12 years and 17 years. Participants were not analysed according to race.

### Co-morbidities

Of the 111 participants, 92 (83%) had at least one co-morbid condition. The most common co-morbidity was cognitive impairment, reported in 46 cases (41%). The second most prevalent co-morbidity was ASD (18%), followed by ODD (11%). Several participants presented with more than one co-morbid diagnosis (see Table 2). Only 19 participants (17%) had a diagnosis of ADHD without any co-morbidities. The authors did not test for differences in co-morbidities linked to age or gender.

**TABLE 2:** Co-morbidities/other diagnoses in all participants (*N* = 101).

Co-morbidity	*N*	%
Cognitive impairment	48	42
ASD	20	18
ODD	12	11
Epilepsy	4	4
Conduct disorder	3	3
Learning disability	3	3
PTSD	3	3
Behavioural issues, not otherwise specified	2	2
Previous meningitis	2	2
Anxiety	1	1
Asperger’s syndrome	1	1
Depression	1	1
Ex-preterm infant	1	1
OCD	1	1

ASD, autism spectrum disorder; ODD, oppositional defiant disorder; PTSD, post-traumatic stress disorder; OCD, obsessive compulsive disorder.

### Standards compliance

Evaluation of compliance with the 10 core audit standards revealed good adherence in 5 standards, fair adherence in 1 and poor adherence in the remaining 4. Of the 6 additional audit standards, 3 demonstrated good compliance, 1 showed fair compliance and 2 showed poor compliance (See Box 1 for details).

BOX 1 Overall compliance to National Institute of Clinical Excellence guidelines.

**Table:** 

Variable		Frequency	Compliance (%)	Compliance Rating
**Core Audit Standards**				
1.	Assessed by a suitably qualified ADHD diagnostician	109	98	GOOD
2.	DSM-5 criteria documented	101	91	GOOD
3.	Specification of ADHD severity	39	35	POOR
4.	Full history and physical exam before diagnosis	108	97	GOOD
5.	Assessment of possible comorbidities	107	96	GOOD
6.	Comprehensive treatment plan (including psychological and/or behavioural and educational intervention)	82	74	FAIR
7.	Methylphenidate offered as appropriate first line treatment	108	97	GOOD
8.	Referral to ADHD parent group	0	0	POOR
9.	Written psycho-education to patients	21	19	POOR
10.	Written psycho-education to caregivers	32	29	POOR
**Additional Audit Standards**				
11.	Patient age, gender documented	111	100	GOOD
12.	Point of view of child documented	34	31	POOR
13.	Contact with the teacher	79	71	FAIR
14.	Growth chart plotting/monitoring	111	100	GOOD
15.	Side effect monitoring	102	92	GOOD
16.	Treatment response monitoring on standard scales	15	14	POOR

*Source*: Adapted from Vrba K, Vogel W, de Vries PJ. Management of ADHD in children and adolescents: clinical audit in South African setting. J Child Adolesc Ment Health. 2016;28(1):1–19

Note: Good = > 80%; Fair = 50 – 79%; Poor = < 50%.

ADHD, attention deficit hyperactivity disorder; DSM-V, Diagnostic and Statistical Manual of Mental Disorders, 5th Edition.

The mean compliance score was 6.37 out of 10 (s.d. = 1.34) for the core audit standards, 4.06 out of 6 (s.d. = 0.91) for the additional standards and 10.44 out of 16 (s.d. = 1.79) for the full set of compliance criteria.

## Discussion

### Demographics and co-morbidities

This study aimed to audit the diagnosis, co-morbidities and management of children and adolescents with ADHD at two hospitals in the Buffalo City Municipality, using the NICE guidelines as the benchmark for best practice, as also recommended by the existing South African ADHD guidelines during that time.^17,26^

Consistent with findings from international audits, most patients were male, aligning with the widely reported male predominance in ADHD diagnoses, which is thought to reflect both biological factors and referral biases.^1,8^ It is likely that girls are underdiagnosed in our setting, a trend commonly observed in ADHD research.^27^ Girls more frequently present with the inattentive subtype of ADHD, which is less disruptive and therefore more easily overlooked by parents, teachers and healthcare providers.^28^

Most of our patients were diagnosed with at least one co-morbid condition (see Table 2). The most common co-morbidity was intellectual disability, followed by ASD and ODD. While this finding aligns with trends reported in other studies, it is notable that intellectual disability was the most frequently observed co-morbidity in this cohort. Typically, ODDs or ASDs are reported as more common co-morbidities in the literature.^15,16,21,29^

South Africa has a high prevalence of learning difficulties, which tend to increase throughout early childhood.^23,25^ One study found that by the age of eight, the mean cognitive development score among children in the Western Cape province of South Africa is approximately 73, which is significantly lower than the global norm of 100.^30^ The Eastern Cape province remains one of the most under-resourced regions in South Africa, facing substantial challenges in both the health and education services, which adversely impact children from birth through to matriculation.^31,32^

### Compliance with audit standards

Nearly all of the patients were diagnosed by a paediatric registrar or paediatrician, and in most cases, the DSM-5 criteria were applied in the diagnostic process. High levels of compliance were observed in the documentation of full patient histories, assessment for co-morbidities prior to diagnosis, comprehensive physical examinations and growth monitoring through plotting of weight and height. These findings reflect the strengths of paediatric training, which emphasises the importance of obtaining thorough medical, social, economic and psychological histories, as well as conducting detailed physical examinations of new patients.

A study conducted in Australia found that paediatricians demonstrate higher adherence to the NICE guidelines for ADHD diagnosis compared to general practitioners. Similarly, the audit conducted at Red Cross War Memorial Children’s Hospital in Cape Town reported lower compliance with physical examinations and growth monitoring in clinics where medical officers, rather than paediatricians, were responsible for patient care. This contrast likely explains the high level of compliance with these specific criteria in our study, as the NDCs are managed by senior paediatric registrars or paediatricians, whose training and clinical approach emphasise comprehensive assessment and documentation.^21^ However, the severity of the diagnosis was documented in the minority of cases. This is a concerning finding, as therapeutic decisions are influenced by both the child’s age and the severity of ADHD at the time of diagnosis. Accurate specification of severity is essential for guiding appropriate treatment strategies and for monitoring response over time.^2,26^

A comprehensive treatment plan was documented in 74% of cases following diagnosis. This included a referral to a psychologist, occupational therapist or speech therapist. However, it is important to consider the context of a low-resource setting, where access to psychological services is often severely limited or, in some cases, entirely unavailable.^23^

In such contexts, alternative interventions may be necessary to support children with ADHD. Non-governmental organisations (NGOs) that support children and their families with mental health challenges do exist, but they are scarce in the Eastern Cape province. One example is *Waves for Change*, an NGO that offers free surf therapy to children with mental health challenges in the Buffalo City Municipality. Similarly, the *Goldilocks and the Bear Foundation* offers free screening and assessments for children with ADHD in underprivileged communities in the Western Cape province (https://waves-for-change.org/surf-therapy/, https://gb4adhd.co.za/). These approaches, where non-specialist health workers such as nurses, social workers or trained community health workers deliver basic behavioural interventions under supervision, have shown promise in improving access to care. Group-based psycho-education sessions for parents and caregivers are being implemented in the hospitals to increase understanding of the condition and improve home-based behavioural management.

Methylphenidate was offered as first-line treatment in most of the cases, consistent with current guidelines and reflecting its status as the only available pharmacological option for ADHD in the public healthcare sector. Good compliance was observed in the documentation of side effects and general treatment response following initiation of treatment. However, the use of standardised rating scales to monitor treatment response was notably poor, with compliance recorded at only 14%. The implementation of standardised monitoring tools should be prioritised, as they provide an objective, structured means of assessing treatment efficacy, as also recommended in the new South African ADHD guidelines.^17^ Such tools also enhance continuity of care, allowing any clinician involved in follow-up to readily understand the patient’s treatment progress without needing to review extensive prior notes.

None of the cases were referred to an ADHD parent support group, as such a service does not currently exist at either Hospital. In addition, only a minority of patients and caregivers were documented as having received written psycho-education. While ADHD information pamphlets are widely available in the clinics, this low rate may reflect a lack of documentation rather than a true absence of distribution. Nonetheless, clear documentation of psycho-education is essential to ensure continuity of care and to confirm that families have been adequately informed about the condition and its management.

Clinicians contacted teachers in the majority of cases; however, there was poor compliance in explicitly exploring the child’s subjective point of view about their behaviour or being on treatment. This is in stark contrast to the Cape Town audit, which showed fair compliance to this audit standard. One possible contributing factor in our setting may be the language barrier between clinicians and patients, which can hinder meaningful communication. Although translators are available at both clinics, their use may not be consistently integrated into consultations. Addressing this gap is essential, as incorporating the child’s voice is a key component of patient-centred care, particularly in the context of neurodevelopmental disorders.

Lost and duplicate folders resulted from malfunctioning hospital administration processes, and files were only excluded when, besides the script, no other information at all was available. This absence of data would not bias the study, as this was because of hospital maladministration. The paediatric outpatient department has since digitalised and is now folder-less to rectify these administrative obstacles.

### Limitations of the current study

As this was a retrospective folder review, data collection relied entirely on the accuracy and completeness of clinical documentation. Some results may have been affected by illegible handwriting and missing information within the folders. Some patients had to be excluded as there was only a prescription and no other information at all in their folder. In addition, the findings of this audit cannot be generalised to the broader paediatric population of the Eastern Cape, as the study was conducted in an urban and periurban setting at a regional and a tertiary-level public hospital. The results also do not reflect practices in the private healthcare sector, where patients often have access to more comprehensive resources and specialist services.

## Conclusion

This audit assessed the adherence of healthcare providers to the NICE guidelines for diagnosis and management of children and adolescents with ADHD. In addition, the demographics and co-morbidities of the children and adolescents with ADHD were described. The findings demonstrated good compliance with diagnostic and clinical management standards. However, the audit also highlighted significant gaps in psycho-social interventions, caregiver support and the availability of structured support groups. These findings underscore the need for systemic improvements and resource allocation beyond pharmacological treatment. The lack of documented psycho-social support and psycho-education highlights the need for more collaboration with ADHD support groups, ADHD NGOs and healthcare workers in the private and public sectors, where these exist. There is an ongoing need to provide further training for healthcare professionals, educators and social workers in the holistic management of ADHD, even more so in a resource-limited environment, where skilled professionals are rare.

This study may serve as a foundation to inform and influence government policy and healthcare planning, particularly regarding the development of auxiliary services and multidisciplinary support for children with ADHD and their families in resource-limited settings.
